# Notch3 and Hey-1 as Prognostic Biomarkers in Pancreatic Adenocarcinoma

**DOI:** 10.1371/journal.pone.0051119

**Published:** 2012-12-04

**Authors:** Christopher D. Mann, Christopher Bastianpillai, Christopher P. Neal, Muhammad M. Masood, Donald J. L. Jones, Friederike Teichert, Rajinder Singh, Elena Karpova, David P. Berry, Margaret M. Manson

**Affiliations:** Department of Cancer Studies and Molecular Medicine, University of Leicester, Leicester, Leicestershire, United Kingdom; National University of Ireland Galway, Ireland

## Abstract

In order to achieve a better outcome for pancreatic cancer patients, reliable biomarkers are required which allow for improved diagnosis. These may emanate from a more detailed molecular understanding of the aggressive nature of this disease. Having previously reported that Notch3 activation appeared to be associated with more aggressive disease, we have now examined components of this pathway (Notch1, Notch3, Notch4, HES-1, HEY-1) in more detail in resectable (n = 42) and non-resectable (n = 50) tumours compared to uninvolved pancreas. All three Notch family members were significantly elevated in tumour tissue, compared to uninvolved pancreas, with expression maintained within matched lymph node metastases. Furthermore, significantly higher nuclear expression of Notch1, -3 and -4, HES-1, and HEY-1 (all p≤0.001) was noted in locally advanced and metastatic tumours compared to resectable cancers. In survival analyses, nuclear Notch3 and HEY-1 expression were significantly associated with reduced overall and disease-free survival following tumour resection with curative intent, with nuclear HEY-1 maintaining independent prognostic significance for both outcomes on multivariate analysis. These data further support a central role for Notch signalling in pancreatic cancer and suggest that nuclear expression of Notch3 and its target gene, HEY-1, merit validation in biomarker panels for diagnosis, prognosis and treatment efficacy. A peptide fragment of Notch3 was detected in plasma from patients with inoperable pancreatic cancer, but due to wide inter-individual variation, mean levels were not significantly different compared to age-matched controls.

## Introduction

Pancreatic ductal adenocarcinoma (PDAC) is associated with poor prognosis due to late presentation and aggressive metastatic potential. The ability to determine prognosis more accurately for individual patients would guide surgical and chemotherapeutic treatment. Suitable biomarkers could help to detect the disease much earlier. To date, prognostic markers of survival have included surgery-related factors and tumour characteristics, including size, perineural and microvascular invasion, local lymph node metastases, resection margin and differentiation status. Serum markers CA19.9, C-reactive protein and neutrophil-lymphocyte ratio (NLR) have also been employed.

Molecular markers associated with prognosis include tumour suppressors, apoptotic proteins, growth factors and receptors, metalloproteinases and angiogenic factors (reviewed in [1]). These have been identified from resected tissues or biopsies. However, biomarkers in blood may allow monitoring of disease progression or response to chemotherapy, as well as aiding earlier diagnosis or screening.

During pancreas development, Notch signalling maintains a population of undifferentiated precursor cells [2]. Mice lacking the downstream effector, HES-1, displayed severe pancreatic hypoplasia caused by depletion of epithelial precursors [3]. The same pathway, up-regulated in PDAC [4]–[6], is involved in tumour development. Notch signalling mediated the tumour-initiating effects of TGF-α by expanding a population of undifferentiated precursor cells and in human PDAC, up-regulation of Notch1, and -4 and Jagged-1 and -2 proteins in resected specimens, as well as PanIN lesions, was observed [5]. HES-1 expression was also significantly increased in metaplastic ductal epithelium, PanIN and invasive PDAC.

Buchler *et al* [4] demonstrated up-regulation of Notch2 mRNA in pancreatic cancer cell lines, along with expression of Jagged ligands. RT-PCR and immunohistochemistry of resected specimens revealed increased Notch3 and -4, Jagged-2 and Delta-1. Notch1 was over-expressed in the nerves; Notch2 and -3 in vascular smooth muscle, and Notch4 in vascular endothelium. Jagged-1 showed high expression at sites of invasion into nerves and surrounding tissue. Transfection with cleaved, active Notch1 (ICN-1) and Jagged-1 resulted in increased VEGF, and Jagged-1 significantly increased tumour cell invasion. These findings implicate Notch in both invasion and angiogenesis.

Wang *et al*. [7] demonstrated high levels of Notch1 in three pancreatic cancer lines. Down-regulation using siRNA caused growth inhibition, G_0_/G_1_ arrest and apoptosis, while increasing Notch1 by transfection significantly promoted cell growth. Notch1 knockdown also inhibited invasion, whereas transfection with ICN-1 cDNA had the opposite effect [8]. More recently downregulation of Notch2 was associated with a partial reversal of epithelial-mesenchymal transition [9]. Using the *Kras^G12D^* mouse model [10], Mazur *et al*. [11] reported that Notch2 ablation inhibited PanIN progression and prolonged survival, with animals eventually succumbing to anaplastic pancreatic cancer.

Oncogenic properties of Notch include inhibition of apoptosis [12]–[14], increased proliferation [15], [16] and epithelial-mesenchymal transition [9], [17], [18]. Notch may also be tumour suppressive in certain situations [19], [20]. Notch pathway activation is associated with clinical outcome in various solid tumours, including transitional cell carcinoma of the bladder [21], breast [22], lung [23], ovarian [24], prostatic [25], and renal cell carcinoma [26]. In this study we have correlated Notch signalling with clinical parameters for PDAC.

Identification of a cleavage site (S4) within the transmembrane domain of Notch1 to produce a Notch1 Aβ-like peptide [27] provides the potential to detect pathway activation *in vivo*. The cleavage is mediated by the same presenilin-dependent γ-secretase complex responsible for liberation of the intracellular domain which modulates transcription in the nucleus, and should therefore correlate with activity. However, although a Notch1 peptide has been identified in mouse, secretion of peptides following S4 cleavage of other Notch receptors has not been investigated. We hypothesised that a peptide would be secreted into the blood following cleavage of the Notch3 receptor, and that, as a result of overexpression, it would be detected in the plasma of patients with PDAC. If so it could provide a minimally invasive biomarker.

## Materials and Methods

### Ethics Statement

Ethical committee approval (REC 7176) was obtained from the Directorate of Research and Development, University Hospitals of Leicester UK to use archival tissue and fresh specimens, including blood, obtained from patients with PDAC undergoing surgery or biopsy, identified from the University Hospitals of Leicester MDT database. Clinical data were obtained from patient case notes and computerised records. Patient survival was documented as of 1^st^ January 2011. No patient was lost to follow-up. All patients providing fresh samples also provided written informed consent. Since this was not possible for archival tissues as most patients were deceased at the time of the study, the ethics committee specifically waived the need for consent for these and associated case notes. All patient samples were analysed anonymously.

### Resected group

Forty-two patients who underwent potentially curative resection between October 2000 and May 2007 were included in this study. Patients with ampullary tumours and distal cholangiocarcinomas were excluded. PDAC tissue was available from all patients, surrounding uninvolved pancreatic tissue was available from 35 patients, and involved lymph nodes from 16 patients. Clinicopathological data are displayed in Table 1.

**Table 1:** Clinicopathological data for patients undergoing resection.

	Number (%)	Median (range)
Gender		
male/female	24 (57.1)/18(42.9)	
Age (years)		64 (30–80)
Preoperative Variables		
CA 19–9 (U/ml)		320 (3–10,000)
Bilirubin (µmol/L)		146 (5–410)
Serum ALP (IU/L)		258 (39–6881)
Serum ALT (IU/L)		91 (18–474)
WCC (×10^9^/L)		7.5 (3.8–20.6)
Lymphocytes (×10^9^/L)		1.5 (0.7–3.0)
Neutrophils (×10^9^/L)		5.0 (2.0–16.2)
NLR		3.7 (1.1–11.9)
Albumin (g/L)		36 (29–47)
Creatinine (µmol/L)		72 (49–139)
Operative intervention		
Pancreaticoduodenectomy	36 (85.7)	
Distal pancreatectomy	4 (9.5)	
Total pancreatectomy	2 (4.8)	
Tumour characteristics		
Tumour diameter (mm)		29 (10–50)
Differentiation: well/moderate/poor	4 (9.5)/19(45.2)/18(42.9)	
Nodal status: +ve/−ve	22 (52.4)/19(45.2)	
Tumour stage: 1A/1B/2A/2B/3	1(2)/5(12)/14(33)/21(50)/1(2)	
Microvessel invasion: yes/no	14 (33.3)/25(59.5)	
Perineural infiltration: yes/no	28 (22.7)/12(28.6)	
Resection margin: +ve/−ve	15 (35.7)/27(64.3)	

ALP- alkaline phosphatase, ALT- analine transaminase, NLR –, neutrophil/lymphocyte ratio, WCC – white cell count.

### Unresectable group

Fifty patients diagnosed with unresectable PDAC between January 2003 and January 2007 were also included. Twenty-one (42%) male and 29 (58%) female had a median age of 68.4 years (range 43.9–80.9 years). Twenty-six were unresectable based on locally advanced disease or vascular involvement and therefore tissue was obtained from the pancreatic primary tumour. Another 24 had metastatic disease and of these, 14 provided tissue from liver metastases, 8 from peritoneal disease and 2 from distant lymph nodes.

### Immunohistochemistry

Following histological assessment, formalin-fixed tissues were stained using the EnVision^+^ system-HRP (DAB) kit (DAKO) and serial sections compared for the different antigens which were retrieved by microwaving in Tris EDTA buffer. Conditions were optimised for each antibody (Table 2). Antibodies were from Santa Cruz (Notch1 (C-20) sc-6014; Notch3 (M-134) sc-5593; Notch4 (H-225) sc-5594; HES-1 (H-140) sc25392); or Abcam (HEY-1 (ab22614). Positive tissue controls were included for each antibody (Table 2), along with appropriate negative controls. Specificity of the antibodies was checked by siRNA knockdown and western blots in the case of Notch 1–4 and by western blots for HES and HEY. Slides were counterstained with haematoxylin. Staining was assessed in tumour tissue and ductal epithelium of uninvolved surrounding pancreas. Cytoplasmic staining was assessed semiquantitatively, scoring in 10% increments according to the percentage of tumour cells/ductal epithelial cells stained. Nuclear staining was considered positive if ≥10% of tumour/ductal epithelial cell nuclei stained positive. Surrounding fibrous and inflammatory tissue was not scored. Slides were graded independently by two observers blinded to the clinical data. In cases with >10% discrepancy, slides were re-assessed by both observers simultaneously and a final score agreed.

**Table 2:** Optimised conditions for immunohistochemistry.

Antibody	Dilution	Incubation duration	Positive control tissue
Notch-1	1:1000	4°C overnight	Human epidermis
Notch-3	1:500	RT 1 hour	Mouse kidney
Notch-4	1:500	4°C overnight	Human kidney
HES-1	1:500	4°C overnight	Human liver
HEY-1	1:1000	4°C overnight	Human lung

### Mass spectrometric analysis of Notch3 peptide

Plasma samples from healthy control and colorectal carcinoma patients were obtained from University Hospitals Leicester clinical trial (UHL number 10598). Samples from additional consented age-matched volunteers and PDAC patients were collected with permission from the local Research Ethics Committee.

#### Solid Phase Extraction

Oasis® HLB columns (Waters, 30 mg, 1cc) were mounted on a vacuum manifold maintained at a pressure of approximately 20kPa. Samples were mixed with an equal volume of 4% phosphoric acid, and left on ice for 1 h. Columns were conditioned with 1 ml of HPLC grade methanol, and washed with 1 ml of ultrapure water, before application of samples. After further washing, samples were eluted using 1ml of 40% acetonitrile in 0.1% trifluoroacetic acid (TFA). Eluates were reduced by evaporation for 40 min, freeze-dried and stored at −80°C. Stored samples were reconstituted in 100 µl 50 mM ammonium bicarbonate, pH 7.4, prior to analysis.

#### Size-exclusion filtration

Microcon® filters (10,000 MWt cut-off, Millipore Ltd, Watford, UK) were washed twice with 500 µl ultrapure water for 20 min. Samples were centrifuged for 45 min (14000 g at 4°C), then stored at −20°C until required for immunoprecipitation.

*Notch3 peptide and antibody* The Notch3 peptide and corresponding polyclonal antibody (raised in rabbit) were commissioned from Davids Biotechnologie GmbH (Regensburg, Germany). The peptide sequence for Notch3 was derived from knowledge of the Notch1 receptor S4 cleavage site and the published Notch1 Aβ-like peptide sequence [27].

Notch1 – VQS ETV EPP PPA QLH FMY VAA AAF VLL FFV GCG * V.

Notch3 – VRG EPL EPP EPS VPL LPL LVA GAV LLL VIL VLG * V.

The sequence used to raise the antibody is underlined and * denotes the conserved cleavage site.

#### Immunoprecipitation of Notch3 peptide

A 100 µl aliquot of *Dynabead*® solution (Invitrogen Ltd Paisley, UK) was washed twice in 500 µl citrate-phosphate buffer pH 5.0 with 0.01% Tween 20, before being incubated with the anti-Notch3 antibody for 2 h. Beads were washed 3x with citrate-phosphate buffer, and once with 1ml triethanolamine pH 8.2. Antibody was cross-linked to the beads using 1 ml 20 mM dimethyl pimelimidate in triethanolamine. Beads were washed once with 1 ml 50 mM Tris buffer pH 7.5, then 3x with 0.01% Tween in PBS. Samples were incubated with the beads overnight at 4°C. The beads were washed 3x with 50 mM ammonium bicarbonate pH 7.4 and the peptide was subsequently eluted using 30 µl of 0.1 M citric acid pH 1.5.

#### Mass spectrometric analysis

Prior to analysis, samples were acidified with an equal volume (5 µL) of 0.1% TFA and mixed 1∶1 with matrix (10 mg/ml α-cyano-4-hydroxy-cinnamic acid dissolved in acetonitrile/methanol [1∶1, v/v] containing 0–001%TFA). A 1 µL aliquot of each sample was spotted onto the MALDI target plate. Analysis of the samples was conducted using a MALDI-TOF mass spectrometer (MALDI R, Waters Ltd., Manchester, UK) operated in positive ionisation mode and spectra were acquired over a mass range of 500–3000Da. An adrenocorticotropic hormone (ACTH) fragment 18–39 (Sigma, Poole, UK) was incorporated into the matrix as the internal standard (major ion at 2466 *m/z*) with 25fmol/ spot. For each sample spectra were obtained for four separate spots on the target plate and the ion intensity for the Notch3 peptide averaged. Mass spectrometric data were processed using MassLynx software (version 4.1).

### Statistics

Statistical analyses were performed using SPSS 18.0® (Chicago, USA). For associations between different proteins, percentages of positive staining were compared using Spearman's correlation coefficient. Chi-squared and Fisher's exact tests were used to analyse categorical data, the Mann-Whitney U test to compare continuous variables between independent groups, with the Wilcoxon test for continuous variables between related groups. Prognostic significance of variables was determined by univariate Cox regression analysis, Kaplan-Meier analysis and application of the log-rank test. Multivariate analysis was performed using all variables with p<0.10 on univariate analysis, through their entry into a Cox proportional hazards regression analysis using a stepwise backward procedure. Statistical significance for all tests was defined as p<0.05.

## Results

### Expression of Notch signalling components in resected patient tissues

Tumour areas and uninvolved pancreas were scored for both nuclear and cytoplasmic expression of Notch pathway constituents (Table 3). Cytoplasmic Notch1, -3 and -4 were identified in all resected specimens. Notch2 was not assessed because of lack of a reliably validated antibody.

**Table 3:** Expression of Notch pathway constituents in normal pancreas, resectable and advanced PDAC.

Marker	Location		Resectable disease		Advanced disease		
		Uninvolved pancreas (n = 35)	Pancreatic tissue (n = 42)	Local lymph nodes (n = 16)	Overall (n = 50)	Locally advanced (n = 26)	Metastatic (n = 24)
**Notch1**	N n(%)	0 (0.0%)	11 (26.2%)*	6 (37.5%)*	35 (70%)*†	16 (61.5%)*†	19 (79.2%)*†
	C %(range)	27.5% (0–50%)	75% (15–100%)*	50% (25–95%)*	30% (5–95%)*†	35% (15–90%)*†	45% (5–65%)*†
**Notch3**	N n(%)	0 (0.0%)	20 (47.6%)*	10 (62.5%)*	45 (90%)*†	23 (88.5%)*†	22 (91.7%)*†
	C %(range)	30% (0–85%)	45% (5–85%)*	45% (30–90%)*	45% (15–90%)*	40% (15–85%)*	65% (20–90%)*δ
**Notch4**	N n(%)	0 (0.0%)	8 (19%)*	3 (18.8%)*	34 (68%)*†	18 (69.2%)*†	16 (66.7%)*†
	C %(range)	20% (0–50%)	70% (20–95%)*	45% (20–90%)*	40% (5–85%)*†	45% (5–80%)*†	45% (15–85%)*†
**HES-1**	N n(%)	8 (22.9%)	33 (78.6%)*	12 (75%)*	50 (100%)*†	26 (100%)*†	24 (100%)*†
	C %(range)	45% (5–85%)	50% (20–95%)	45% (5–95%)	40% (15–95%)†	42.5% (15–90%)	40% (15–95%)
**HEY-1**	N n(%)	0 (0%)	11 (26.2%)*	7 (50%)*	38 (76%)*†	18 (69.2%)*†	20 (83.3%)*†
	C %(range)	25% (0–45%)	65% (35–100%)*	70% (45–100%)*	70% (50–100%)*	75% (55–100)*	70% (50–100%)*

* p<0.05 compared to uninvolved pancreas; † p<0.05 compared to resectable PDAC tissue; δ p<0.05 compared to locally advanced disease.

N  =  nuclear; C  =  cytoplasmic; n  =  number.

#### Notch1

Cytoplasmic expression was increased in tumour compared to surrounding uninvolved pancreatic tissue in 28/35 patients (80.0%) for whom both tissues were available. Nuclear Notch1, indicative of pathway activation, was not identified in normal ducts, but occurred in tumours of 11 patients with resected PDAC (26.2%, p<0.001). Notch1 was positively correlated with HES-1 expression in tumour cytoplasm (p = 0.020) and nuclei (p = 0.013), but was not associated with any clinicopathological factors listed in Table 1 (data not shown).

#### Notch3

**Figure 1:**
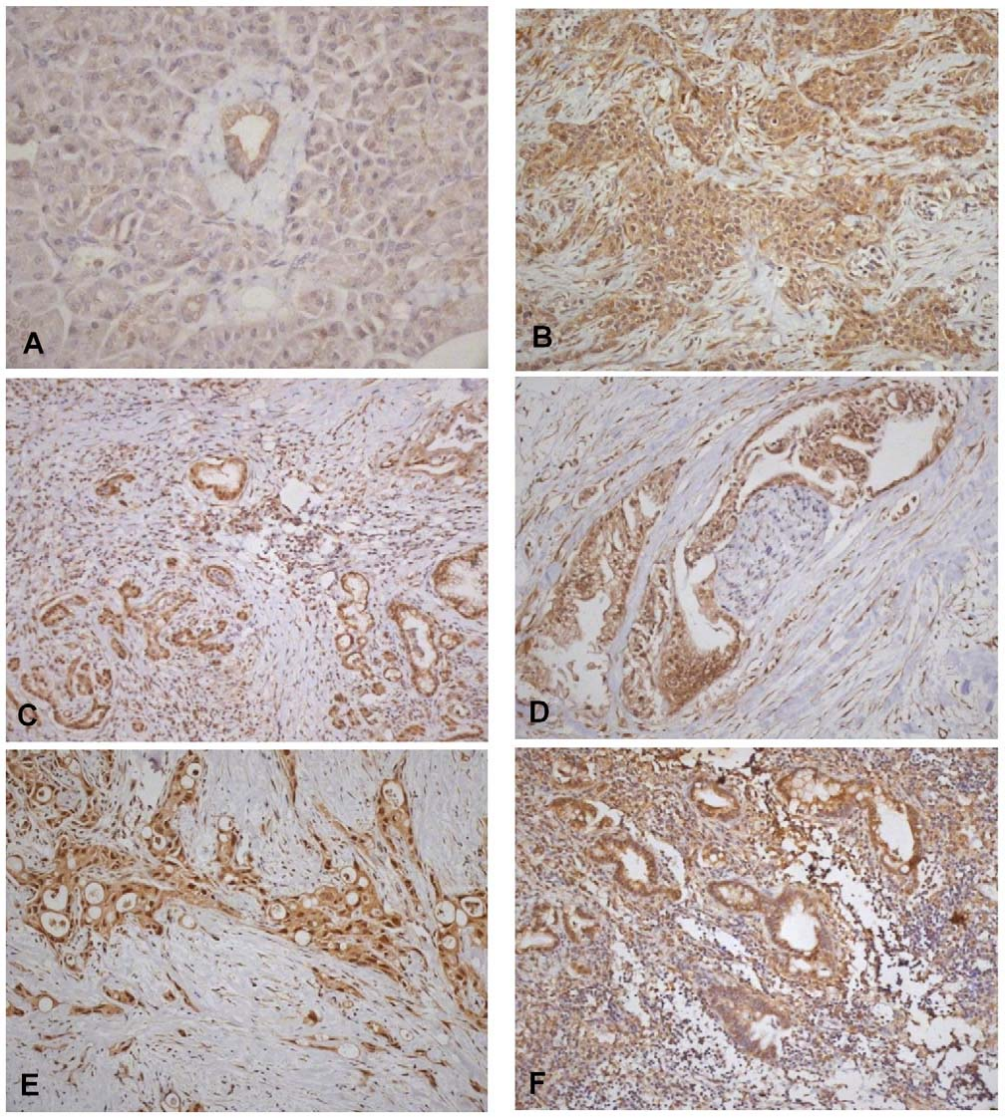
Notch3 in normal pancreas and PDAC. a) Notch3 in the cytoplasm of normal pancreatic ductal epithelial cells; b) resected PDAC demonstrating strong cytoplasmic Notch3 with some positive nuclei; c) resected PDAC demonstrating largely nuclear stain; d) nuclear stain in an area of perineural invasion; e) strong cytoplasmic and nuclear stain in an unresectable specimen; f) Notch3 in a metastatic deposit in a local lymph node. (Original magnification a – 40x, b-f – 20x).

Cytoplasmic expression was up-regulated in tumours in 21/35 patients (60.0%). Nuclear Notch3 was absent in normal ductal cells, but present in 20 resected PDACs (47.6%, p<0.001 compared with uninvolved pancreas) (Table 3, Figure 1). Notch3 expression correlated with HEY-1 in cytoplasm and nucleus of resected specimens (p = <0.001). Nuclear Notch3 was associated with the presence of lymph node metastases in resected PDAC specimens (p = 0.015) and there was a trend towards association with tumour stage (p = 0.07), but not with other clinicopathological features.

#### Notch4

Cytoplasmic expression was up-regulated in tumours in 33/35 patients (94.3%). Nuclear Notch4 was absent from normal ductal cells, but present in 8 resected PDAC (Table 3). Significant inverse associations were noted between Notch4 and Notch1. Cytoplasmic Notch4 was also positively associated with nuclear Notch3 (p = 0.009). Notch4 was not associated with any clinicopathological variable, although there were trends towards increased cytoplasmic expression associated with well/moderate tumour differentiation and negative lymph node status (p = 0.086 and 0.050 respectively).

#### HES-1

Cytoplasmic HES-1 was detected in all resected specimens, with up-regulation in 8/35 patients (22.9%). Nuclear stain was identified in normal ductal cells of 8 patients (22.9%), but in the tumours of 33 resected patients (78.6%, p<0.001 Table 3). HES-1 was significantly associated with Notch1 (p = 0.020 cytoplasmic; 0.013 nuclear), but not with any clinicopathological variable, although there was a trend towards nuclear expression being associated with perineural invasion (p = 0.073).

#### HEY-1

**Figure 2:**
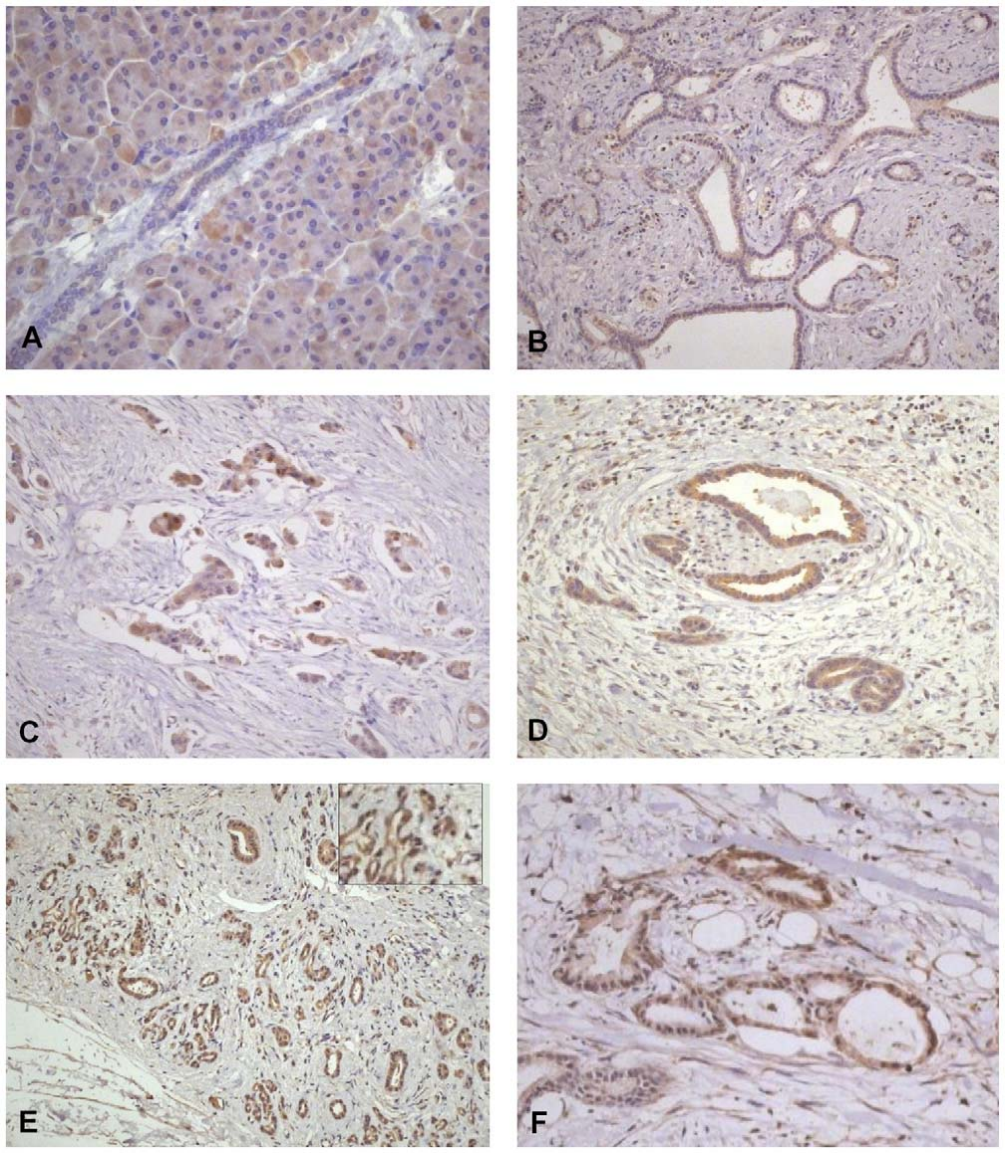
Hey-1 in normal pancreas and PDAC. a) Normal pancreas showing faint cytoplasmic stain for Hey-1; b) resected PDAC demonstrating cytoplasmic, but no nuclear, staining; c) resected PDAC demonstrating nuclear expression; d) area of perineural invasion in a resected tumour demonstrating cytoplasmic staining; e) unresectable tumour demonstrating cytoplasmic and positive nuclear expression of HEY-1; f) peritoneal metastases demonstrating cytoplasmic and nuclear staining. (Original magnification 20x, except a and inset 40x).

In contrast, HEY-1 was up-regulated in tumours from 33/35 patients (94.2%), with nuclear stain in 11 resected PDAC (26.2%, p = 0.001) and 38 (76%) advanced tumours (Table 3; Figure 2). There were significant associations between HEY-1 and Notch3 (p≤0.001). Nuclear HEY-1 in PDAC was strongly associated with the presence of local lymph node metastases and microvessel invasion (p = 0.003), as well as perineural invasion (p = 0.048), younger age (p = 0.022), and a trend towards association with tumour stage (p = 0.058).

### Biomarkers in local lymph node metastases

Increased expression of all biomarkers was maintained in local lymph node metastases of resectable tumours, with HEY-1 showing a trend towards higher nuclear expression here than in the primary tumour (50.0% vs. 26.2%, p = 0.074) (Table 3).

### Biomarker expression in advanced PDAC

In tissues from inoperable locally advanced and metastatic tumours, nuclear expression of Notch1, -3, -4, HES-1, and HEY-1 (all p≤0.001) was significantly increased compared to expression in resected tumours (Table 3). Cytoplasmic Notch1, Notch4 and HES-1 were significantly reduced in advanced PDAC compared to resected tumours, with no difference in cytoplasmic staining of Notch3 (p = 0.953) or HEY-1 (p = 0.945) between the two groups.

When patients with metastatic disease were compared to those with locally advanced disease, only cytoplasmic expression of Notch3 differed significantly between the groups, being higher in those patients with distant metastases (p = 0.018).

### Outcome

For patients undergoing resection, median follow-up was 30.6 months (mean 40.1 months, range 1–122 months). As of January 2011, seven patients (16.7%) were alive, four of whom were disease-free, two died of unrelated illnesses and thirty-six (88%) had developed recurrent disease. Median overall survival was 30.5 months (95% CI 29.5–31.5 months), with 1-, 3- and 5-year overall survival of 87.5%, 32.5% and 21.3%. Median disease-free survival was 25.0 months (95% CI 17.7–32.3 months), with 1-, 3-, and 5-year rates of 64.1%, 25.6% and 16.6%. In patients with unresectable tumours, median overall survival was 5.9 months (95% CI 3.4–8.4 months), with a 17.0% 12-month survival rate (p<0.0001 vs. resectable disease).

### Univariate analyses of biomarkers with respect to survival

**Figure 3:**
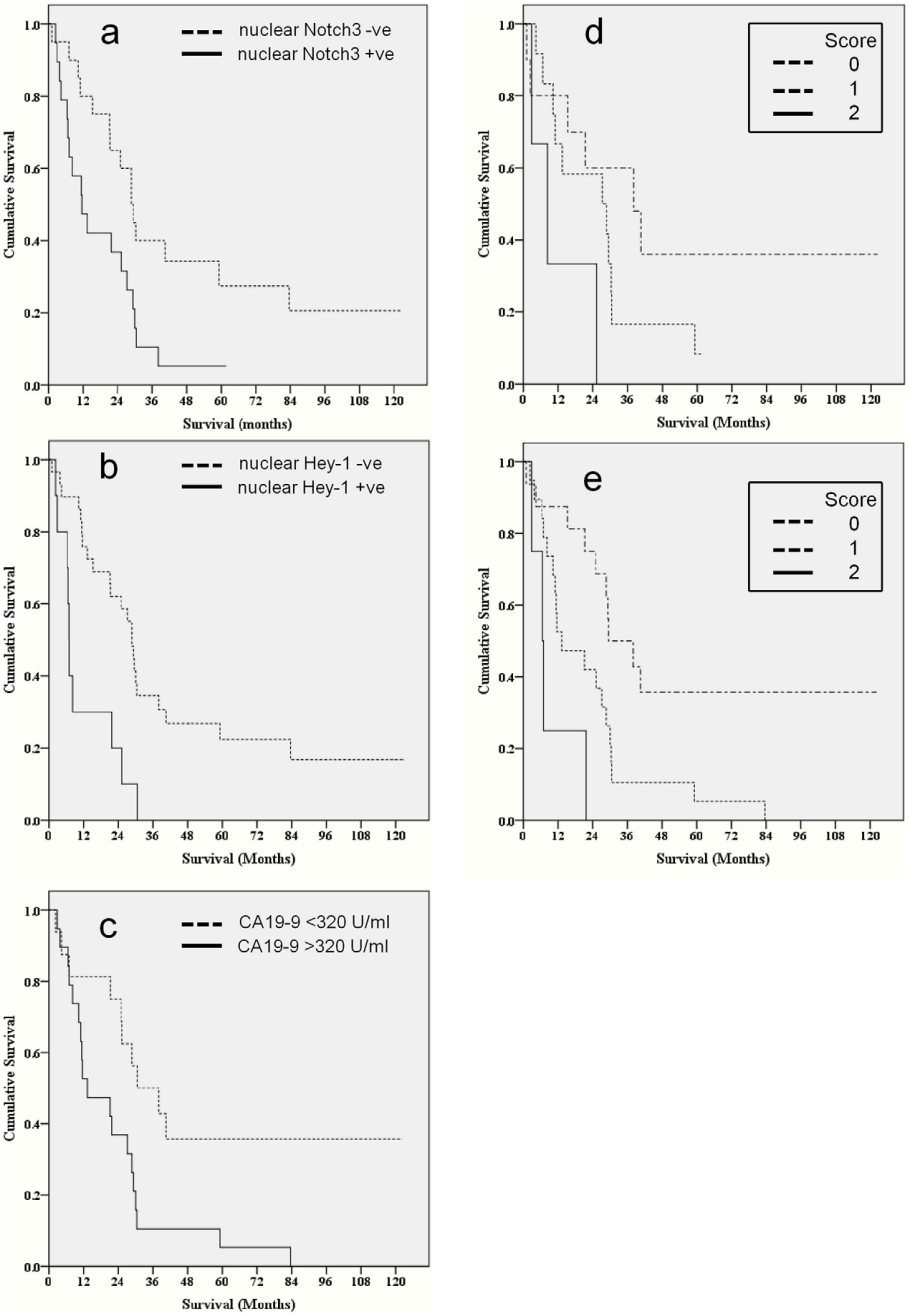
Impact of Notch-3, Hey-1 and CA19-9, and combined biomarker expression on disease-free survival. Kaplan-Meier survival curves demonstrating the impact of a) nuclear Notch-3 expression (p = 0.014); b) nuclear Hey-1 (p = 0.001); c) serum CA19.9 (p = 0.010) on disease-free survival. Kaplan-Meier survival curves demonstrating the impact of a score based upon CA 19.9 level and nuclear HEY-1 expression disease-free survival for d) all patients (p<0.001) and e) patients undergoing a R_0_ resection (p = 0.041).

Biomarker expression was related to overall and disease-free survival following resection (Table 4). On univariate analysis, nuclear Notch1 was associated with significantly shorter overall survival (p = 0.044, log-rank test), but not disease-free survival. Nuclear Notch3 expression was also associated with significantly worse prognosis following resection, where the 5-year overall survival was 5.0% (median 27.3 months, 95% CI 20.7–33.9 months) compared with 38.6% for patients without nuclear expression (median 32.1 months, 95% CI 24.5–39.8 months, p = 0.011; Figure 3). Similarly the 5-year disease-free survival was 5.3% (median 11.7 months, 95% CI 4.3–18.9 months) compared to 27.4% (median 28.7 months, 95% CI 27.0–30.5 months, p = 0.014). Notch-4 had no significant impact on survival rates.

**Table 4:** Univariate Cox regression survival analyses for patients undergoing resection.

	Overall Survival		Disease-free Survival	
	Hazard Ratio (95%CI)	P	Hazard Ratio (95%CI)	P
Gender: male/female	0.702 (0.351–1.404)	0.317	0.913 (0.457–1.825)	0.797
Age: ≥64/<64 years	0.899 (0.447–1.809)	0.766	0.972 (0.489–1.933)	0.936
CA19.9 ≥320/<320 (U/ml)	2.828 (1.288–6.212)	**0.010**	2.743 (1.239–6.069)	**0.013**
Tumour diameter: ≥29/<29 (mm)	0.839 (0.407–1.729)	0.634	0.845 (0.412–1.730)	0.644
Tumour differentiation: poor vs. well/moderate	1.286 (0.616–2.687)	0.503	0.985 (0.477–2.033)	0.967
Nodal status: +/−	3.441 (1.535–7.578)	**0.003**	3.438 (1.566–7.548)	**0.002**
Tumour stage: 1/2/3	3.164 (1.226–8.165)	**0.017**	2.370 (1.005–5.593)	**0.049**
Microvessel invasion: +/−	1.716 (0.921–3.584)	0.100	1.822 (0.874–3.799)	0.109
Perineural invasion: +/−	1.723 (0.890–3.758)	0.098	2.208 (1.001–4.872)	**0.050**
Resection margin: positive/negative	1.536 (1.075–2.193)	**0.018**	1.431 (0.881–2.160)	0.081
Notch1 nuclear expression: +/−	2.128 (1.003–4.525)	**0.049**	1.658 (0.781–3.521)	0.188
Notch1 cytoplasmic expression: ≥50%/<50%	0.626 (0.311–1.261)	0.190	0.700 (0.349–1.407)	0.317
Notch3 nuclear expression: +/−	2.541 (1.213–5.324)	**0.013**	2.380 (1.166–4.858)	**0.017**
Notch3 cytoplasmic expression: ≥50%/<50%	1.516 (0.760–3.023)	0.238	1.768 (0.868–3.600)	0.116
Notch4 nuclear expression: +/−	1.155 (0.445–2.998)	0.768	0.898 (0.345–2.340)	0.826
Notch4 cytoplasmic expression: ≥50%/<50%	0.550 (0.274–1.103)	0.092	0.596 (0.298–1.190)	0.142
HES-1 nuclear expression: +/−	1.252 (0.514–3.053)	0.621	1.444 (0.553–3.769)	0.453
HES- 1 cytoplasmic expression: ≥50%/<50%	0.689 (0.317–1.498)	0.347	0.595 (0.273–1.296)	0.191
HEY-1 nuclear expression: +/−	2.998 (1.326–6.778)	**0.008**	3.498 (1.583–7.690)	**0.002**
HEY-1 cytoplasmic expression: ≥50%/<50%	0.803 (0.307–2.100)	0.654	0.676 (0.260–1.763)	0.424

With regard to downstream targets of Notch, expression of HES-1 showed no significant association with overall or disease-free survival. However, nuclear HEY-1 was highly associated with shorter survival. The 5-year overall survival was 0.0% (median 26.5 months, 95% CI 11.9–41.2 months) compared with 28.4% for patients without nuclear expression (median 31.3 months, 95% CI 25.5–37.1 months, p = 0.006 Figure 3). The 5-year disease-free survival was also 0.0% (median 7.1 months, 95% CI 6.4–7.7 months) compared to 22.3% in patients without nuclear expression (median 28.7 months, 95% CI 25.1–32.4 months, p = 0.001).

### Multivariate analyses of Notch biomarkers with respect to survival

On multivariate analysis, in addition to CA19.9 level and resection margin status, nuclear HEY-1 maintained independent prognostic significance for both overall (p = 0.006) and disease-free survival (p = 0.001) (Table 5). Nuclear Notch-3 expression also demonstrated a non-significant trend towards shortened overall survival (p = 0.053). When only patients who underwent a R_0_ resection (negative resection margin) were considered (Table 5), nuclear HEY-1 expression maintained independent significance for both overall (p = 0.022) and disease-free survival (p = 0.005), along with serum CA19.9 level.

**Table 5:** Relevance of biomarkers to survival in PDAC.

	Overall Survival		Disease-free Survival	
	Hazard Ratio (95% CI)	P value	Hazard Ratio (95% CI)	P value
**all resections (n = 42)** Resection margin: positive/negative	3.197 (1.299–7.868)	**0.011**		
CA19.9 ≥320/<320 (U/ml)	3.034 (1.292–7.125)	**0.011**	3.539 (1.532–8.176)	**0.003**
Notch3 nuclear: +/−	2.671 (1.030–6.928)	0.053		
HEY-1 nuclear: +/−	3.591 (1.441–8.946)	**0.006**	4.147 (1.724–9.977)	**0.001**
**R0 resections (n = 27)**				
CA19.9≥320/<320 (U/ml)	2.432 (1.106–6.275)	**0.066**	5.323 (1.699–16.677)	**0.004**
Notch3 nuclear: +/−	4.271 (0.787–8.834)	0.124	2.380 (0.816–6.942)	0.112
HEY-1 nuclear: +/−	4.164 (1.224–14.161)	**0.022**	5.545 (1.693–18.157)	**0.005**

Multivariate Cox regression survival analyses following resection (results from the final round of regression analysis).

### Prognostic significance of combining biomarkers

Combinations of biomarkers are likely to provide more accurate prognostic information. Nuclear HEY-1 expression and serum CA19.9 levels were chosen due to their independent prognostic power on multivariate analysis for all patients (disease-free survival) and those having undergone a R_0_ resection. Patients were scored for the presence of serum CA19.9 level ≥320 U/ml and positive nuclear HEY-1 expression, with a minimum score of 0 and a maximum of 2. When all resected tumours were analysed, this score was significantly associated with both overall and disease-free survival (Table 6; Figure 3).

**Table 6:** Prognostic significance of combined biomarkers.

Score	5yr Overall Survival			5yr Disease-free Survival		
All resections	% patients	Median (months)	95% CI p = 0.001	% patients	Median (months)	95% CI p<0.001
0	43.8	49.3	21.3–77.3	38.1	38.1	24.2–52.0
1	10.0	30.1	25.1–35.1	5.3	21.2	1.7–40.8
2	0.0	26.5	7.9–45.2	0.0	7.1	6.2–8.0
**R_0_ resections**			p = 0.007			p = 0.041
0	46.7	49.3	46.2–53.1	36.0	38.1	12.4–63.8
1	15.4	31.7	30.5–33.0	8.3	27.2	1.2–53.2
2	0.0	27.3	12.2–42.5	0.0	8.3	0.0–16.7

Scores: 0 =  negative for nuclear HEY-1, CA19.9<320 U/ml; 1 =  positive for nuclear HEY-1 **or** CA19.9>320 U/ml; 2 =  positive for nuclear HEY-1 **and** CA19.9>320 U/ml; CI, confidence interval.

### Detection of Notch3 peptide in plasma

**Figure 4:**
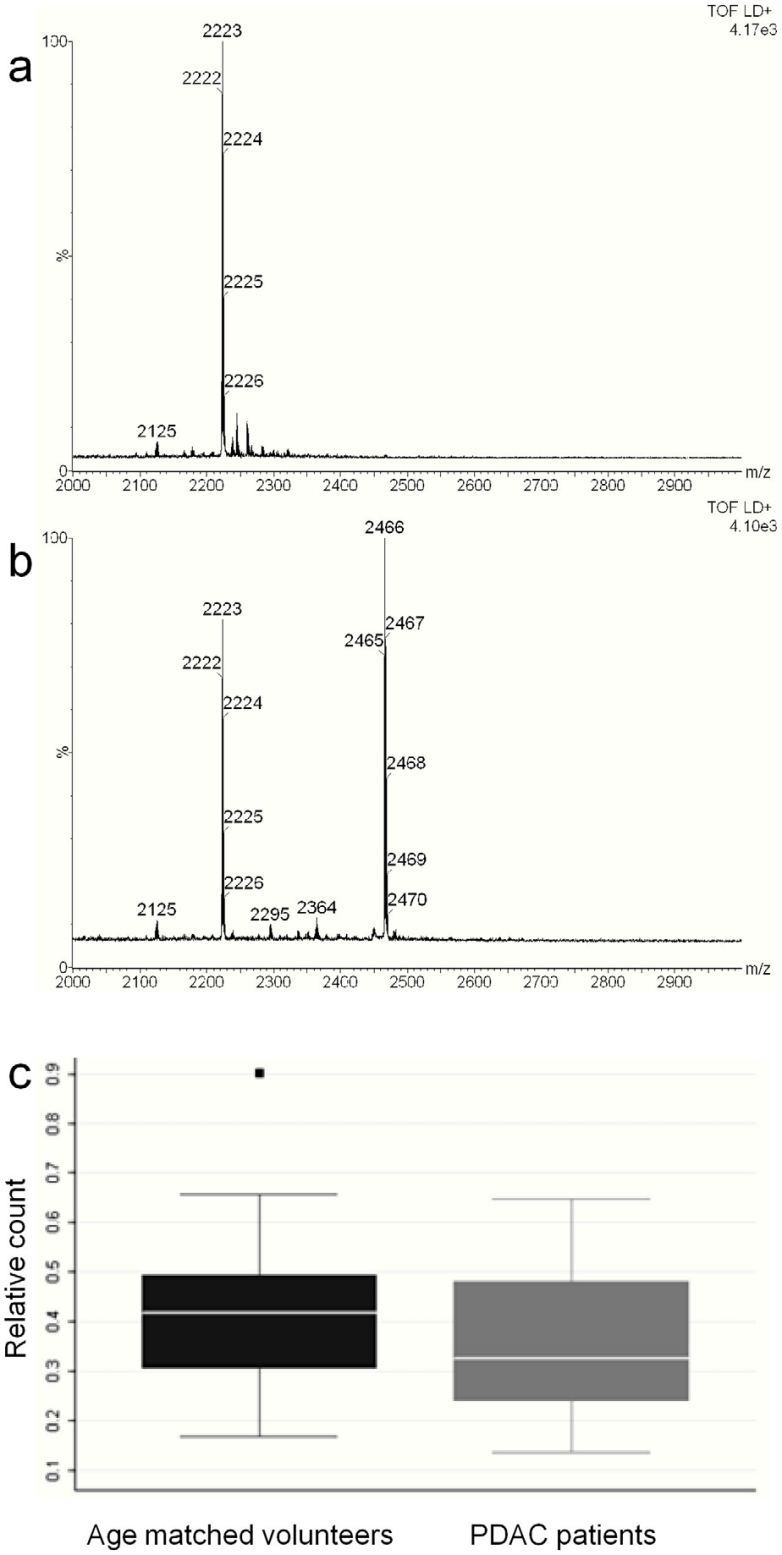
Mass spectrometric analysis of the Notch3 peptide. Mass spectra of a) the Notch3 peptide standard showing the major ion at 2223 *m/z*; b) an eluted immunoprecipitate from a healthy plasma sample, indicating presence of the Notch3 peptide (the ion at 2466 *m/z* corresponds to the internal standard, ACTH). c) Levels of Notch3 peptide in volunteer (n = 31) and patient (n = 31) samples. Age matched volunteers: mean  = 0.420± SD  = 0.155; median  = 0.426 (0.168–0.901); outlier: 0.901. PDAC patients: mean  = 0.348± SD  = 0.145; median: 0.326 (0.137–0.648), Outliers: none.

Because of the possible prognostic significance of Notch3 signalling described above, we investigated its potential as a plasma biomarker in PDAC. MALDI-TOF mass spectrometric analysis of the Notch3 peptide standard resulted in a major ion at 2223 *m/z* (Figure 4a), with a limit of detection of 5 fmol/μl. Plasma samples from age-matched volunteers (55–77 yrs, n = 31) and non-resectable PDAC patients (n = 31) were analysed semiquantitatively for the presence of Notch3 peptide. To determine whether Notch3 expression was specific for PDAC, samples from patients with primary (n = 14) and metastatic (n = 15) colorectal cancers were also included. An ion with 2223 *m/z* corresponding to the Notch3 peptide was detected in the majority of samples (Figure 4b), such that the mean ion intensity in cancer patients was not significantly higher than in volunteers (Figure 4c). Similar levels of the peptide were also detected in patients with primary and metastatic colorectal cancer (data not shown).

## Discussion

We carried out a comprehensive IHC assessment of Notch pathway components in primary PDAC, advanced local and metastatic disease and correlated expression patterns with clinical parameters to assess their prognostic significance. To our knowledge, this is the first time this has been done in pancreatic cancer.

Up-regulation of Notch1, -3 and -4, along with HES-1 and HEY-1, had been observed previously in resected PDAC [5] and particularly in advanced disease. Those findings agreed with Buchler *et al* [4] who demonstrated strong immunostaining of Notch receptors and ligands in areas of neurovascular invasion. In the present study, nuclear expression of Notch1 and HES-1 as well as Notch3 and Hey-1 were positively correlated. This supports previous data suggesting that HES-1 is predominantly under Notch1 regulation, whilst Notch3 is thought to act through HEY-1 [28]–[30]. Notch3 was shown to be a poor activator of HES-1 and HES-5, and even to block Notch1-mediated activation of HES promoters *in vitro* [31]. Notch3 and -4 are structurally divergent from Notch1 and -2, lacking the transactivation domain in the cytoplasmic portion of the receptor [32]. Nuclear Notch1 was found to be negatively correlated with cytoplasmic Notch4, as was nuclear Notch4 with cytoplasmic Notch1, suggesting a negative feedback relationship or antagonism between these two receptor pathways.

Nuclear Notch3 expression was associated with the presence of local lymph node metastases in resected PDAC specimens. Similarly, nuclear HEY-1 expression was associated with the presence of nodal metastases, and perineural and microvascular invasion. These data suggest that Notch3 signalling through HEY-1 is associated with a more aggressive tumour phenotype.

We found that on multivariate analysis, nuclear HEY-1 is independently predictive of poor overall and disease-free survival, suggesting it merits further investigation as a prognostic biomarker. Such biomarkers may inform on novel therapeutic targets and design of adjuvant therapies following surgery. Nuclear Notch3 expression was also included in the multivariate survival analysis, but did not quite reach significance. However, interestingly, if nuclear HEY-1 was excluded from the multivariate analysis, nuclear Notch3 became independently predictive of outcome for both overall and disease-free survival for all patients (p = 0.007 and 0.048) and those undergoing R_0_ resections (disease-free survival only; p = 0.030). Thus Notch3, acting via HEY-1, also appears to be associated with poor prognosis. We previously reported that nuclear Notch3 expression was associated with nonresectability [6]. Notch3 has been shown to be important in several solid tumours, with gene amplification (19p13.12) detected in breast [33] and ovarian carcinoma [34]. It has also been associated with lymph node metastatasis and poor prognosis in ovarian carcinoma [24], and resistance to carboplatin chemotherapy [35]. Notch3 signalling has been reported to prevent apoptosis by cross-talk with the MAPK pathway and regulation of Bim [36], as well as by acting through the proto-oncogene, Pbx1 [37].

Our data clearly demonstrate a progressive up-regulation of the Notch pathway in primary PDAC, which continues in advanced disease. We also show for the first time that activity of Notch3 in particular, through its target gene, HEY-1, is associated with an aggressive tumour phenotype and an adverse prognosis.

The predictive power of isolated molecular biomarkers is limited, so combining them with clinical and pathophysiological data may lead to a more robust and accurate assessment of cancer prediction and prognosis. We showed that nuclear HEY-1 expression combined with CA 19.9 levels can be used to create a score which allows stratification of prognosis following resection of PDAC. These and other molecular markers now need to be validated in larger cohorts of PDAC patients to develop a highly reliable panel of biomarkers for prognosis.

Since Notch3 is upregulated in PDAC, we hypothesised that a peptide fragment might be released into patient blood. Based on published data for murine Notch1, we predicted the peptide sequence and commissioned an antibody suitable for immunopreciptation. Analysis of healthy plasma samples revealed that the Notch3 peptide was present. On further analysis of colorectal carcinoma and PDAC patient samples the peptide was detected in the majority of samples from all cohorts. Statistical analysis revealed no difference between the levels observed in cancer patient cohorts and volunteers. These results provide evidence for the first time that a Notch3 peptide is released into blood in humans following S4 cleavage of this receptor. Unfortunately it does not appear to be a specific biomarker for PDAC. It is, however, possible that Notch3 peptide levels in plasma, sampled before and after treatment in the same individual could provide a measure of treatment efficacy.

Expression is significant in most individuals and this may be explained by its presence in vascular smooth muscle cells (VSMC) where activation of the pathway was shown to propagate cell cycle progression, with Notch3 upregulation linked to vascular injury [38]. Mutations in Notch3 are associated with CADASIL (cerebral autosomal dominant arteriopathy with subcortical infarcts and leukoencephalopathy), a condition that causes recurrent strokes and vascular dementia due to VSMC degeneration [39]. Thus Notch3 has an important role in the homeostasis of arterial VSMCs [40], promoting their survival and preventing apoptosis and migration [41], [42].

In summary, this study has identified nuclear expression of Notch3 and HEY-1 in tumours as potentially useful indicators of poor prognosis in PDAC. Assessing nuclear HEY-1 expression in combination with CA19.9 levels could help to predict outcome for patients undergoing resection.
